# State of Charge Estimation and Evaluation of Lithium Battery Using Kalman Filter Algorithms

**DOI:** 10.3390/ma15248744

**Published:** 2022-12-07

**Authors:** Longzhou Hu, Rong Hu, Zengsheng Ma, Wenjuan Jiang

**Affiliations:** School of Materials Science and Engineering, Xiangtan University, Xiangtan 411105, China

**Keywords:** lithium battery, state of charge, adaptive, Kalman filter algorithms

## Abstract

The accurate and rapid estimation of the state of charge (SOC) is important and difficult in lithium battery management systems. In this paper, an adaptive infinite Kalman filter (AUKF) was used to estimate the state of charge for a 18650 LiNiMnCoO_2_/graphite lithium-ion battery, and its performance was systematically evaluated under large initial errors, wide temperature ranges, and different drive cycles. In addition, three other Kalman filter algorithms on the predicted SOC of LIB were compared under different work conditions, and the accuracy and convergence time of different models were compared. The results showed that the convergence time of the AUKF algorithms was one order of magnitude smaller than that of the other three methods, and the mean absolute error was only less than 50% of the other methods. The present work can be used to help other researchers select an appropriate strategy for the SOC online estimation of lithium-ion cells under different applicable conditions.

## 1. Introduction

Since the 1990s, lithium batteries have become one of the best choices for current consumer-grade electric vehicle power batteries due to their good stability and high energy density. To ensure the safety and reliability of electric vehicles (EVS), the battery management system (BMS) must provide real-time and accurate information about the usage status of the on-board power battery pack. The SOC (state of charge) is one of the most important states tracked in a battery to optimize the performance and extend the lifetime of batteries, so its estimation is an important task for battery management [1,2]. The accurate estimation of the SOC involves many nonlinear effects such as open circuit voltage (OCV), instantaneous current, charge and discharge rate, ambient temperature, battery temperature, parking time, self-discharge rate, Coulomb efficiency, resistance characteristics, SOC initial value, depth of discharge (DOD), etc. [3]. These factors are affected by different materials and processes, and they also interact with each other, so the SOC calculation of power batteries is complex and difficult, which is a challenge that has not been overcome for many years [4].

In recent years, ternary lithium-ion batteries have been considered as power batteries with great application prospects and market competitiveness due to their outstanding specific capacity, good rate performance, and high working voltage [5]. In order to allow the ternary lithium-ion battery to perform better, it is necessary to use appropriate methods to evaluate its SOC. The estimation methods of the SOC are mainly divided into the following categories [6]. (1) The ampere-hour integration method [7], which obtains the battery SOC by integrating the current during the charge and discharge processes in the dimension of time. This method is simple, but it is sensitive to the initial SOC value and is prone to error accumulation. (2) The open-circuit voltage (OCV) method [8], which establishes the relationship between the OCV and SOC based on the off-line open-circuit voltage test [9]. The advantage of this method is its high estimation accuracy, which requires a long standing time to eliminate the influence of the polarization voltage. Therefore, the OVC method is suitable for battery SOC estimation under static equilibrium conditions, [10] but it is difficult to use for online SOC estimation. (3) The machine learning method [11] includes fuzzy logic [12], support vector machines [13], neural networks, [14], and a genetic algorithm [15]. The use of machine learning methods to estimate SOC is worthwhile, but its interpretation is poor, and it usually requires large-scale tests and high experimental costs. (4) The model-based method [16] such as the Kalman filter (KF) [17], particle filter (PF) [18], H-infinity filter [19], and nonlinear observer [20]. Among them, the Kalman filter method is an online algorithm with closed-loop feedback, which has the characteristics of a small calculation amount and easy realization. Its accuracy is greatly improved compared with the ampere-hour method and voltage method, and the calculation cost and time are much lower than that of the machine learning method. Therefore, the Kalman filtering method is more popular than the other three methods and more appropriate for battery management in real-time and BEVS/HEVS control [21]. Therefore, the Kalman filtering method was used to estimate the SOC of ternary lithium-ion batteries in this paper.

Kalman filtering (KF) is an algorithm that uses the linear system state equation to estimate the optimal state of the system of which the inputs are the current and temperature of the cell, and the output is the terminal voltage. For the traditional Kalman filter algorithm, there are usually some problems in the SOC index such as offset, drift, and long-term state divergence, etc. Hence, an extended Kalman filter (EKF) is proposed to counter these problems [22]. The EKF uses the first-order partial derivative of the nonlinear function Taylor expansion (ignoring higher-order terms) to linearize the nonlinear system model to use the KF algorithm for filter estimation [23]. Therefore, if the assumption of local linearity is violated, linearization would cause the filter to be highly unstable. In order to improve the shortcoming of this method, an unscented Kalman filter (UKF) is used to estimate the SOC [24]. Unlike the EKF, the UKF designs a small number of σ points, and calculates the propagation of the first- and second-order statistical characteristics of random vectors by propagating the σ points through the nonlinear functions [25]. Therefore, it can better approximate the nonlinear characteristics of the state equation than the EKF, and thus has higher estimation accuracy than the EKF [26]. However, in the EKF and the UKF, the process noise covariance and measurement noise covariance are set to constant values, which are usually determined in advance by the trial and error method. The disadvantage of these two algorithms is that the trial and error time is too long and error-prone [27]. To solve this problem, an adaptive extended Kalman filter (AEKF) [28] and adaptive unscented Kalman filter (AUKF) [29,30] were proposed to update the process noise covariance and the measurement noise covariance in an adaptive manner to improve the SOC estimation accuracy.

As a suitable SOC estimation method, it not only requires high precision, a fast response, and low computing cost, but can also adapt to different practical application environments. Recently, some new Kalman filtering algorithms have been proposed such as an adaptive dual Kalman filter (ADKF) [31,32,33], a dual extended Kalman filter algorithm (DEKF) [34], a dual unscented Kalman filter algorithm (DUKF) [35], and a multiscale parameter adaptive dual Kalman filter algorithm [36]. The proposed dual-filter algorithms provide a new research method for lithium-ion battery SOC estimation, but they cannot significantly improve the accuracy; the calculation time is also significantly increased, so it is difficult for it to be used for online SOC estimation. Shrivastava [37] et al. proposed a dual forgetting factor-based adaptive extended Kalman filter to solving the shortcomings of the battery model parameter deviation from the true value affecting the estimation accuracy in the dual Kalman filter algorithm. Then, they estimated the battery parameters and SOC using the multi-time scale DEKF to improve the computational efficiency [38]. Xing and Wu [39] presented an improved adaptive unscented Kalman filter that could improve the SOC estimation stability and improve the SOC estimation accuracy by estimating and correcting the system noise statistics adaptively. Aside from Kalman filter algorithms, other methods have also been proposed. Wen [40] et al. provided a multi-state control strategy that could effectively manage the SOC while improving the system frequency stability. A dual-input neural network combining gated recurring unit (GRU) layers and fully connected layers (acronymized as a DIGF network) was developed by taking both the time-series voltage and current measurements and the battery’s SOH as inputs [41]. The present works in the literature have focused more on the accuracy and speed of the algorithms, with less emphasis on the usage environment, which has important effects for successful estimation.

In this paper, considering the actual working environments of an electric vehicle, response speed, and calculation cost, the AUKF was used to estimate their SOC under large initial errors, wide temperature ranges, and different drive cycles. The results were compared with the EKF, UKF, and AEKF to provide a suitable SOC algorithm for designers.

## 2. Establishment of Battery Model

### 2.1. Subjects

The single battery selected in this paper was a 18,650 LiNiMnCoO_2_, graphite lithium-ion cell, and its main parameters are shown in Table 1.

### 2.2. Model Structure

An accurate estimation of the SOC of a battery is strongly dependent on the appropriate battery model. There are three types of commonly used battery models: electrochemical models, black-box models, and equivalent circuit models (ECMs) [42]. Among these models, the ECMs can be analyzed and expressed by a mathematical model, which has a clear physical meaning, in addition to a simple and flexible intuitive structure. The main advantage lies in the convenience of online calculation and computer simulation analysis. Therefore, an equivalent circuit model was used in this paper.

**Figure 1:**
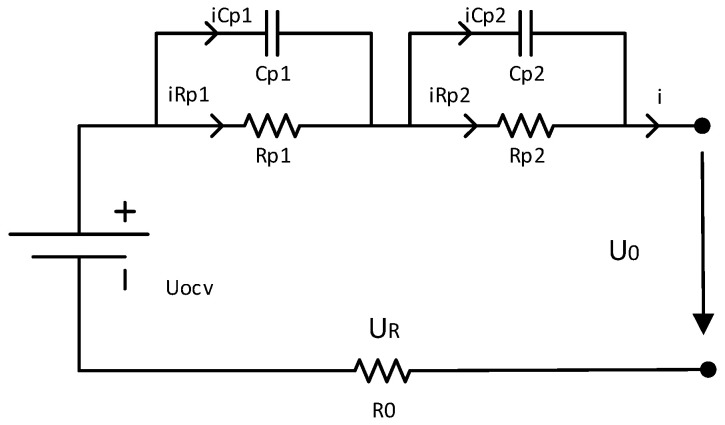
Second-order RC battery model.

The ECMs use traditional circuit elements such as resistance, capacitance, and a constant voltage source to describe the external characteristics of power batteries, which is convenient for battery characteristic analysis and model parameter identification [43]. The higher the equivalent circuit order, the better the data fitting effect and the more complex the model. According to the trade-off between the structural complexity and the model prediction accuracy, a second-order model was selected, as shown in Figure 1.

U_ocv_ is equal to the open circuit voltage (OCV) of the battery which is related to the SOC; *R*_0_ represents the contact resistance between the active material, the current collector, the lead electrode, and the active material/current collector. The dynamic characteristics of the power battery are described by the polarization resistance, *R_pi_*, and the polarization capacitance, *C*_pi_, including the polarization characteristics and the diffusion effect, where *i* = 1, 2; *I* is the charge and discharge current, of which the charge is negative, and the discharge is positive; and *U*_0_ is the terminal voltage of the battery.

In the second-order RC battery model shown in Figure 1, the voltages *V*_1_ and *V*_2_ at both ends of the capacitors C_p1_ and C_p2_ and the *SOC* are the state variables; *I* is the input variable; and *U*_0_ is the output variable. Combined with the ampere-hour integration method [44], the discredited state space model shown in Equation (1) can be obtained. Based on this model, the AUKF was used to estimate the battery SOC.

(1) $$\left[\begin{array}{c} V_{1(k+1)}\\ V_{2(k+1)}\\ SOC(k+1) \end{array}\right]=\left[\begin{array}{c} R_{P1}(1-\exp(-\frac{\Delta t}{R_{P1}C_{P1}}))\\ R_{P2}(1-\exp(-\frac{\Delta t}{R_{P2}C_{P2}}))\\ -\frac{\eta \Delta t}{C_{N}} \end{array}\right]I(k)+\left[\begin{array}{ccc} \exp(-\frac{\Delta t}{R_{P1}C_{P1}}) & 0 & 0\\ 0 & \exp(-\frac{\Delta t}{R_{P2}C_{P2}}) & 0\\ 0 & 0 & 1 \end{array}\right]\left[\begin{array}{c} V_{1(k)}\\ V_{2(k)}\\ SOC_{\left(k\right)} \end{array}\right]$$

where Δ*t* is the sampling interval, taken as Δ*t* = 1 s; *V*_1(*k*)_, *V*_2(*k*)_, and *SOC*_(*k*)_ refer to the voltage across the capacitors *C*_p1_, *C*_p2_, and *SOC* at *k* sampling time; *V*_1(*k*+1)_, *V*_2(*k*+1)_, and *SOC*_(*k*+1)_ refer to the voltage across the capacitors, *C*_p1_ and *C*_p2_, and the *SOC* at *k* + 1 sampling time; *η* is the Coulomb efficiency; *C*_N_ is the total capacity of the battery; and *U*_ocv_ is the open circuit voltage of the battery, which is a function of the SOC.

### 2.3. Introduction of Experimental Platform

**Figure 2:**
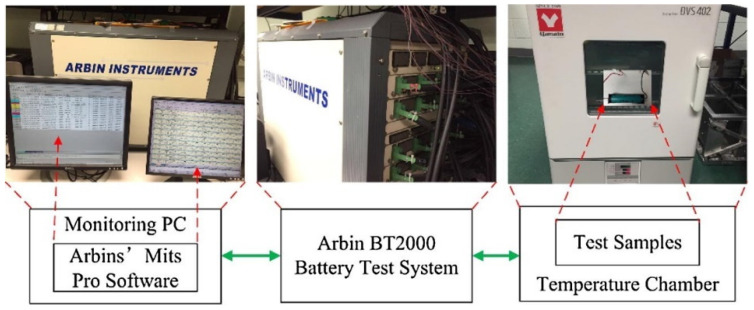
Test platform diagram.

The experimental platform is shown in Figure 2, which was used to obtain the terminal voltage and current of the battery. The PC sends commands to an Arbin BT2000 battery test system to simulate the battery working conditions as well as to charge or discharge the battery. The battery test system transmits the collected voltage and current data to the PC in real-time.

### 2.4. Measurement of the Relationship Curve between OCV and SOC

Obtaining the OCV of the battery is critical because it can be used for resistance-capacitance parameter identification and the SOC estimation. The OCV is a single-valued function of the SOC; therefore, the corresponding OCV can be obtained through the relationship curve between the OCV and SOC. The low-current OCV test and the incremental the OCV test are two common methods for observing the OCV–SOC relationship, while the latter has high tracking accuracy and anti-interference ability [45]; therefore, this paper used the latter to obtain the relationship curve between the OCV and SOC.

**Figure 3:**
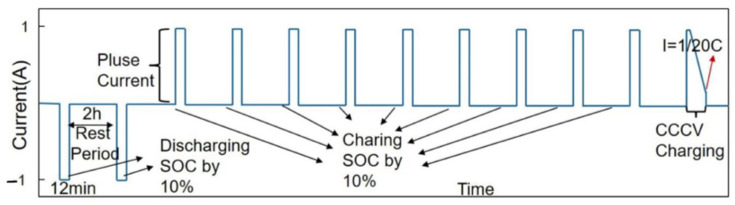
Test profile of the incremental OCV test.

**Figure 4:**
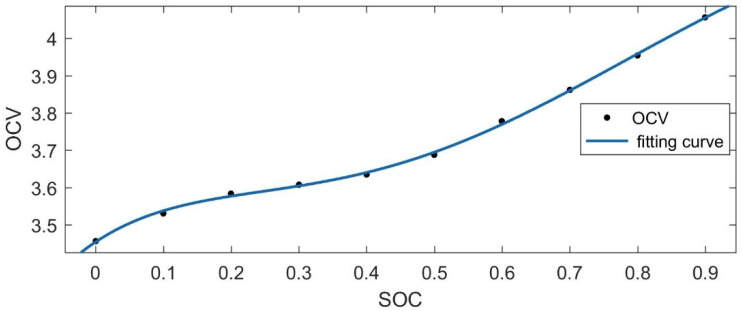
OCV–SOC curve.

The incremental OCV test is shown in Figure 3. First, the cell was discharged until its SOC became 0%. Then, the cell was charged by using a positive pulse current (i.e., C/20) with a width corresponding to a certain amount of charge (i.e., 10% SOC). In the relaxation period, the SOC was allowed to stand for 2 h to eliminate the polarization effects inside the cell. When the terminal voltage rose to the upper cutoff voltage, it entered into the constant voltage charging stage. In this stage, when the charging current dropped below C/20, the charging was completed. Finally, an averaging step and a linear interpolation step were applied to obtain the OCV–SOC curve. The OCV–SOC curve obtained by the linear interpolation method is shown in Figure 4. The curve was fitted into a fifth-order OCV–SOC function using MATLAB, and its OCV–SOC equation is:

(2) $$f(x)=17.31x^{5}-50.64x^{4}+55.47x^{3}-27.15x^{2}+6.16x+3.029$$

### 2.5. Identification of the Resistance-Capacitance Parameters of the Battery Model

#### 2.5.1. Model Parameter Identification Test

**Figure 5:**
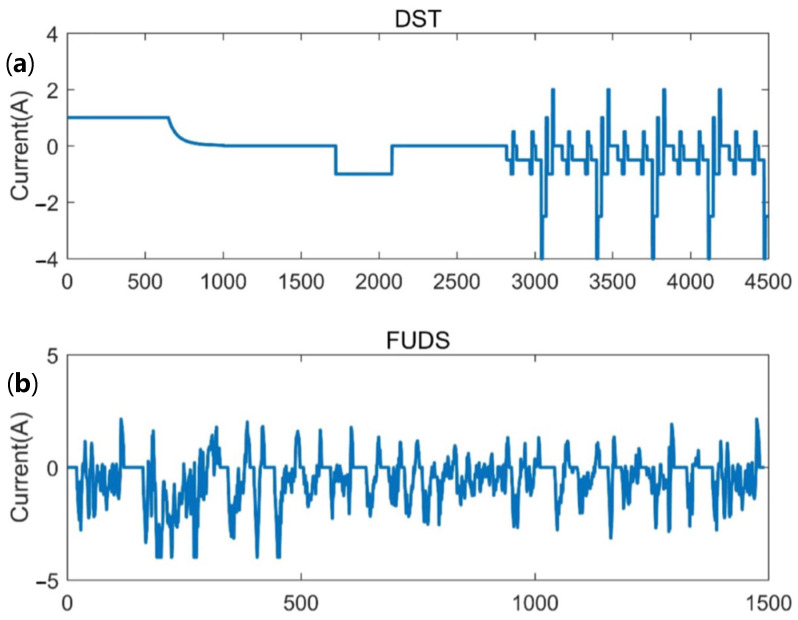
Battery test load curve: (**a**) Dynamic stress test; (**b**) federal urban driving schedules.

In this paper, the first half of the data of the dynamic stress test (DST) was used to identify the model parameters on the test samples. The DST was specified in the USABC (American Advanced Battery Association) test programs as collecting data, simulating the dynamic discharge state, and can be reduced to the utmost required quantity according to the specified performance of the test sample. Figure 5a shows the current section of the DST. Although the DST consists of sorts of current steps with different amplitudes and lengths, and takes into account regenerative charging, it is still a simplification from the actual loading conditions of the battery. Thus, the DST was performed on the test battery to determine the model parameters in this study. In order to evaluate the SOC estimation results (such as the accuracy and robustness), not only the DST, but a more complex dynamic current profile, the federal urban driving schedule (FUDS), was used. Similar to the DST, the current sequence of the FUDS was also transmitted from the time-speed profile of industry standard vehicles. The corresponding current profile is shown in Figure 5b.

#### 2.5.2. Model Parameter Identification Method

The exponential fitting method is used to fit the response curve of the battery terminal voltage to the pulse current, and then the parameters in the battery model can be obtained. The equation of discharge current and output voltage in the circuit model shown in Figure 1 can be expressed as:

(3) $$U_{0}=U_{\mathrm{OCV}}-IR_{0}-IR_{p1}\left(1-\exp\left(-\frac{t}{R_{p1}C_{p1}}\right)\right)-IR_{p2}\left(1-\exp\left(-\frac{t}{R_{p2}C_{p2}}\right)\right)$$

The MATLAB exponential fitting expression used was:

(4) $$U=c_{0}+c_{1}\exp\left(-\lambda_{1}t\right)+c_{2}\exp\left(-\lambda_{2}t\right)$$

where *c*_0_, *c*_1_, and *c*_2_ are constants. Comparing Equations (3) and (4), we obtain:

(5) $$\begin{array}{l} R_{p1}=\frac{c_{1}}{I},R_{p2}=\frac{c_{2}}{I};\\ C_{p1}=\frac{1}{\lambda_{1}R_{p1}},C_{p2}=\frac{1}{\lambda_{2}R_{p2}};\\ R_{0}=\left(U_{\mathrm{OCV}}-c_{0}-c_{1}-c_{2}\right)/I \end{array}$$

**Figure 6:**
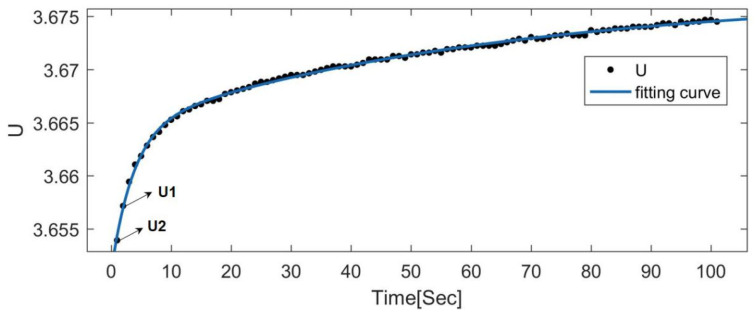
Terminal voltage response curve when the current suddenly reaches 0.

Since the OCV changes little with the SOC in the platform voltage region, resulting in a large error, the following method can be used to obtain *R*_0_:

For Equation (3), $\underset{t\to 0}{\lim}(1-\exp(-\frac{\tau }{R_{P1}C_{P1}}))=0$, and $\underset{t\to 0}{\lim}(1-\exp(-\frac{\tau }{R_{P2}C_{P2}}))=0$; therefore, when *t* approaches 0, Equation (3) can be simplified to $U=U_{\mathrm{OCV}}-IR_{0},$$dU=(U_{\mathrm{OCV}}-U){|}_{instant}=IR.$ Applying it to the actual circuit as shown in Figure 1, the following equation is obtained:

(6) $$R_{0}=\frac{U_{1}-U_{2}}{I}$$

where *U*_2_ and *U*_1_ are the cell terminal voltages of two consecutive sampling points before and after the current suddenly drops to zero (the data used were from 2000 to 2200 corresponding to the abscissa of Figure 5a). Using this method, the terminal voltage response curve when the current suddenly reaches 0 can be obtained, as shown in Figure 6.

Referring to the “Freedom CAR Battery Experiment Manual”, without considering the charging situation, the model parameters as shown in Table 2 were obtained by using Equations (3)–(6) via the discharge method.

## 3. AUKF Algorithm for SOC Estimation

**Figure 7:**
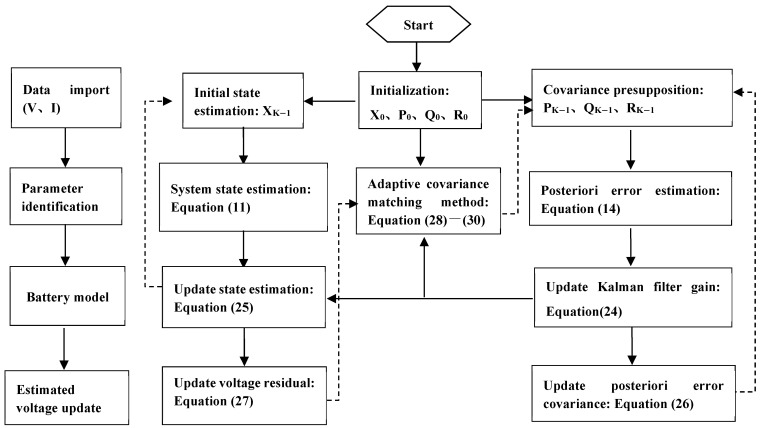
Flowchart of the adaptive unscented Kalman filter algorithm.

For the Kalman filter algorithms, it must be assumed that the noise is Gaussian white noise. However, the statistical characteristics of noise in the actual BMS during data acquisition are unknown. Adopting the adaptive Kalman filter method, the state variables can be dynamically estimated from the measurement data, and the statistical characteristics of noise can be continuously estimated and corrected, so the SOC of the battery can be accurately estimated. The estimation process of the SOC using the AUKF is shown in Figure 7.

The AUKF algorithm for SOC estimation mainly includes four steps, as shown below.

### 3.1. Algorithm Initialization

Initial state estimation:

$$X_{0}={\left[0_{}\;0_{}\;\overset{\wedge }{S_{0}}\right]}^{T}$$

where $\overset{\wedge }{S_{0}\;}$ is the initial SOC value; 

Initial posterior error covariance: *P*_0_;

Initial process noise covariance: *Q*_0_;

Initial measurement noise covariance: *V*_0_;

Window size for covariance matching: *L*.

### 3.2. Prediction

#### 3.2.1. Sigma Points (UT Transform) Are Generated at the *k*−1 Moment

Sigma data point sequence is constructed by using a series of sampling points. The sigma point generated at *k*−1 step can be expressed as:

(7) $${\vec{X}}_{k-1}^{\left(0\right)}={\overset{\wedge }{X}}_{k-1}$$

(8) $${\vec{X}}_{k-1}^{\left(i\right)}={\overset{\wedge }{X}}_{k-1}+{\sqrt{(N+\lambda )P_{k-1}}}_{}i=1,2,\cdots N$$

(9) $${\vec{X}}_{k-1}^{\left(i\right)}={\overset{\wedge }{X}}_{k-1}-{\sqrt{(N+\lambda )P_{k-1}}}_{}i=N+1,\cdots 2N$$

where $\lambda =3\alpha^{2}-1$ is a scale parameter, which can be adjusted to improve the approximation accuracy; *α* is the scaling coefficient, which determines the distribution of sigma points and is generally set to a very small positive value; and *N* is the dimension of the extended state variable.

#### 3.2.2. Time Update

Update the sample point:

(10) $${\bar{X}}_{k|k-1}^{\left(i\right)}=f({\vec{X}}_{k-1})+Q_{k-1}(i=0,...,2N)$$

The prior estimation is obtained:

(11) $${\overset{\wedge }{X}}_{k|k-1}^{}=\sum_{i=0}^{2N}W_{m}^{\left(i\right)}{\bar{X}}_{k|k-1}^{\left(i\right)}$$

where ${\hat{x}}_{K|K-1}$ is the predicted value of the sigma points at the *k*|*k*−1 moment and $W_{m}^{\left(i\right)}$ is the weight used to calculate the mean value, which is determined by the following formula:

(12) $$W_{m}^{\left(0\right)}=\frac{\lambda }{\lambda +N}$$

(13) $$W_{m}^{\left(i\right)}=\frac{1}{2(\lambda +N)}(i=1,2,\cdots 2N)$$

The prior error covariance of the system state is obtained:

(14) $$P_{k|k-1}=\sum_{i=0}^{2N}W_{c}^{\left(i\right)}({\bar{X}}_{k|k-1}^{\left(i\right)}-{\overset{\wedge }{X}}_{k|k-1}^{}){\left({\bar{X}}_{k|k-1}^{\left(i\right)}-{\overset{\wedge }{X}}_{k|k-1}^{}\right)}^{T}+Q_{k-1}$$

where $W_{c}^{\left(i\right)}$ is the weight used to calculate the covariance, which is determined by the following formula:

(15) $$W_{c}^{\left(0\right)}=\frac{\lambda }{\lambda +N}+(1-\alpha^{2}+\beta )$$

(16) $$W_{c}^{\left(i\right)}=\frac{1}{2(\lambda +N)}(i=1,2,\cdots 2N)$$

where the constant *β* is generally determined by experience, and for the Gaussian distribution, it is generally taken as *β =* 2.

### 3.3. Survey Update

#### 3.3.1. Generating Sigma Points at the *k*|*k*−1 Moment

The sigma point generated at *k*|*k*−1 step can be expressed as:

(17) $${\vec{X}}_{k|k-1}^{\left(0\right)}={\overset{\wedge }{X}}_{k|k-1}$$

(18) $${\vec{X}}_{k|k-1}^{\left(i\right)}={\overset{\wedge }{X}}_{k|k-1}+{\sqrt{(N+\lambda )P_{k|k-1}}}_{}\;i=1,2,\cdots N$$

(19) $${\vec{X}}_{k|k-1}^{\left(i\right)}={\overset{\wedge }{X}}_{k|k-1}-{\sqrt{(N+\lambda )P_{k|k-1}}}_{}\;i=N+1,\cdots 2N$$

#### 3.3.2. Calculating the Predicted Output Voltage and Covariance

The predicted output voltage for the sigma points at *k*|*k*−1 step ${\bar{U}}_{{}_{K|K-1}}^{\left(i\right)}$ is calculated by:

(20) $$\begin{array}{l} {\bar{U}}_{k|k-1}^{\left(i\right)}=K_{0}{{\vec{S}}_{k|k-1}^{\left(i\right)}}^{5}+K_{1}{{\vec{S}}_{k|k-1}^{\left(i\right)}}^{4}+K_{2}{{\vec{S}}_{k|k-1}^{\left(i\right)}}^{3}+K_{3}{{\vec{S}}_{k|k-1}^{\left(i\right)}}^{2}\\ +K_{4}{\vec{S}}_{k|k-1}^{\left(i\right)}+K_{5}-V_{1}-V_{2}-RI(i=0.1,\cdots ,2N) \end{array}$$

(21) $${\hat{U}}_{k|k-1}^{}=\sum_{i=0}^{2N}W_{m}^{\left(i\right)}{\bar{U}}_{k|k-1}^{\left(i\right)}$$

The predicted output voltage covariance at the *k*|*k*−1 moment *D_k_* can be expressed as:

(22) $$D_{k}=\sum_{i=0}^{2N}W_{c}^{\left(i\right)}({\bar{U}}_{k|k-1}^{\left(i\right)}-{\hat{U}}_{k|k-1}^{}){\left({\bar{U}}_{k|k-1}^{\left(i\right)}-{\hat{U}}_{k|k-1}^{}\right)}^{T}+V_{k-1}$$

#### 3.3.3. Modifying System State Estimation

The cross-covariance of the state variables and output variables at the *k*|*k*−1 moment is calculated by:

(23) $$E_{k}=\sum_{i=0}^{2N}W_{c}^{\left(i\right)}({\vec{X}}_{k|k-1}^{\left(i\right)}-{\overset{\wedge }{X}}_{k|k-1}^{}){\left({\bar{U}}_{k|k-1}^{\left(i\right)}-{\hat{U}}_{k|k-1}^{}\right)}^{T}$$

where *U_k_* is the measurement of the battery voltage at the *k* moment.

The Kalman gain *G_k_* is calculated by:

(24) $$G_{k}=E_{k}D_{k}^{-1}$$

State estimation correction:

(25) $${\overset{\wedge }{X}}_{k}={\overset{\wedge }{X}}_{k|k-1}+G_{k}(U_{k}-{\hat{U}}_{k|k-1})$$

State covariance correction:

(26) $$P_{k}=P_{k|k-1}-G_{k}D_{k}G_{k}{}^{T}$$

### 3.4. Adjustment of Q and V

In this brief, the adaptive estimation of the process noise covariance *Q* and measurement noise covariance *V* on the basis of the voltage residual of the battery model and the covariance of the voltage residual were considered. Therefore, *Q* and *V* were estimated and updated iteratively from the following

(27) $$\mu_{k}=U_{k}-\left[\begin{array}{l} K_{0}{\hat{S}}_{k}{}^{5}+K_{1}{\hat{S}}_{k}{}^{4}+K_{2}{\hat{S}}_{k}{}^{3}+K_{3}{\hat{S}}_{k}{}^{2}\\ +K_{4}{\hat{S}}_{k}+K_{5}-V_{1}-V_{2}-R_{}I \end{array}\right]\;$$

(28) $$F_{k}\approx \frac{\sum_{n=k-L+1}^{k}\mu_{n}\mu_{n}{}^{T}}{L}$$

(29) $$V_{k}=F_{k}+\sum_{i=0}^{2N}W_{c}^{\left(i\right)}({\bar{U}}_{k|k-1}^{\left(i\right)}-U_{k}+\mu_{k}){\left({\bar{U}}_{k|k-1}^{\left(i\right)}-U_{k}+\mu_{k}\right)}^{T}\;$$

(30) $$Q_{k}=G_{k}F_{k}G_{k}{}^{T}\;$$

where *μ_k_* is the voltage residual of the battery model at the *k* moment, and *F_k_* is the approximation to the covariance of the voltage residual at the *k* moment.

Through the iteration of the above steps 2~4, the AUKF is established. The accuracy of the SOC estimation is improved by adaptive adjustment of the process noise and measurement noise.

## 4. Experimental Results and Analysis

### 4.1. Simulation with Different Initial Errors

**Figure 8:**
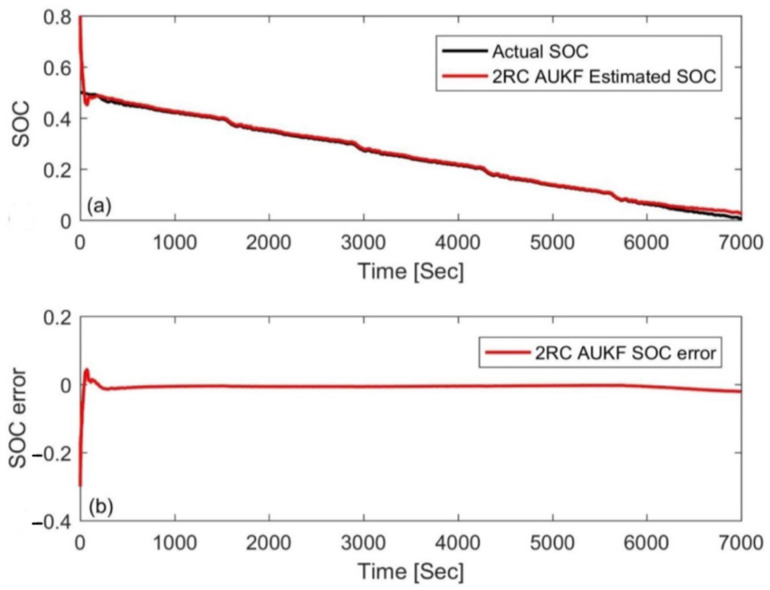
Estimation results by the AUKF at an initial SOC value of 80%. (**a**) SOC estimation comparison curve; (**b**) SOC estimation error.

**Figure 9:**
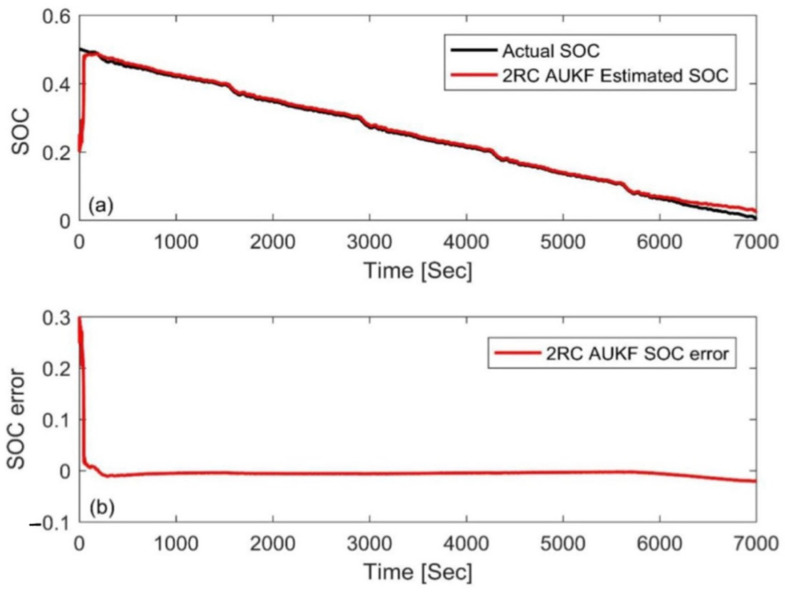
Estimation results by the AUKF at an initial SOC value of 20%. (**a**) SOC estimation comparison curve; (**b**) SOC estimation error.

In this paper, a FUDS cycle was used to test the samples, the temperature was set to 25 °C, and the initial SOC value of the experiment was arbitrarily set to 50%. For verification and comparison, the initial SOC value, *S*_0_, in the simulation was set to 80% and 20%, respectively (the initial error is ±30%). The estimation results based on the AUKF are shown in Figure 8 and Figure 9 in which the reference value of the SOC is calculated by the ampere-hour method. The results show that the AUKF can quickly compensate for the initial SOC error and accurately track the experimental SOC values under different initial values. After correcting the initial error, the difference between the two results was almost indistinguishable. Figure 8b and Figure 9b show that the estimation error was large in the early stage due to inaccurate initial values, but after a certain convergence time, the estimation error was stable, within 2%. In order to evaluate and compare the performance of the algorithm from the two aspects of estimation accuracy and robustness, the mean absolute error (MAE) and convergence rate were used in this paper. The MAE of SOC can be calculated by using the following formula:

(31) $$MAE=\frac{1}{n}\sum_{k=1}^{n}\left|S_{k}-\overset{\wedge }{S_{k}}\right|$$

where *S_k_* is the experimental SOC at time *k*. The convergence rate is the corresponding time when the error converges below 2%

Table 3 shows the MAE and convergence rate under two different SOC initial values. The AUKF corrects the SOC prediction via the real-time online prediction and estimation of noise, quickly adjusts the influence of inaccurate initial values, and causes its estimated value to converge to the actual SOC; therefore, it has good robustness.

### 4.2. Simulation at Different Temperatures

**Figure 10:**
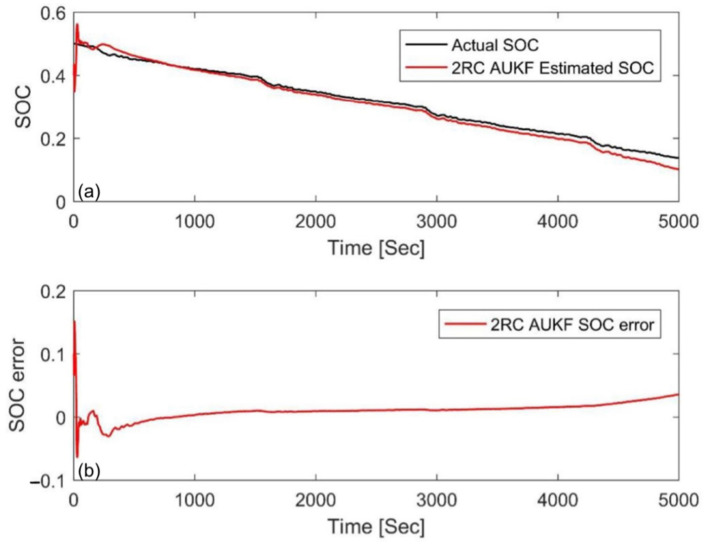
Estimation results at an initial SOC of 40% via the FUDS at 0 °C. (**a**) SOC estimation comparison curve; (**b**) SOC estimation error.

**Figure 11:**
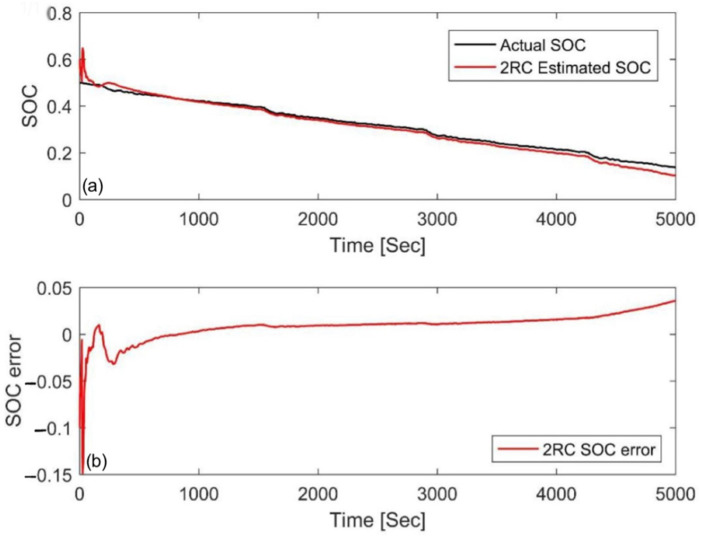
Estimation results at an initial SOC of 60% via the FUDS at 0 °C. (**a**) SOC estimation comparison curve; (**b**) SOC estimation error.

In order to evaluate the performance of the AUKF at different temperatures, Figure 10 and Figure 11 show the estimation results and errors when the temperature is 0 °C and the initial value error is ±10%, respectively. Compared with the results at 25 °C, the initial errors can also be compensated for quickly, but the differences between the stable value and the actual SOC were relatively larger than those at 25 °C. The MAE was 0.0132 and 0.0136, respectively, as shown in Table 4. This indicates that the AUKF exhibited excellent performance at low temperature.

**Figure 12:**
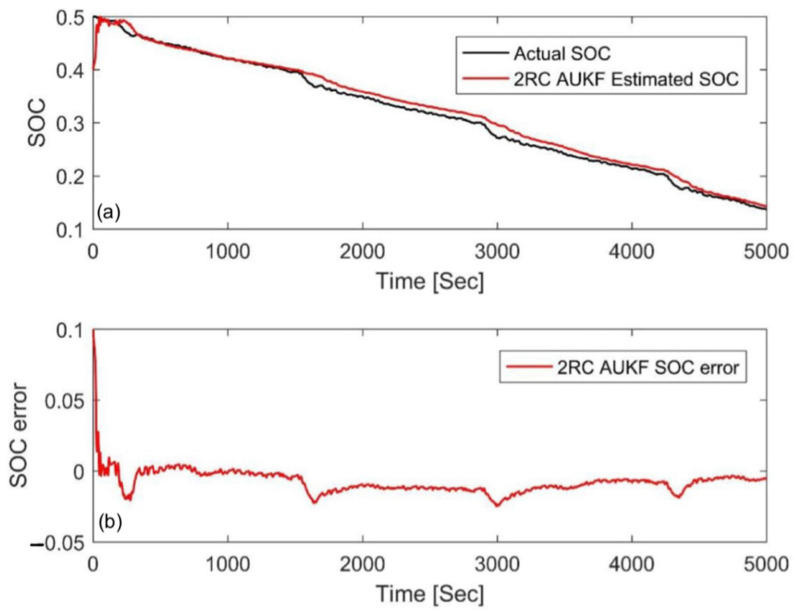
Estimation results at an initial SOC of 40% via the FUDS at 45 °C. (**a**) SOC estimation comparison curve; (**b**) SOC estimation error.

**Figure 13:**
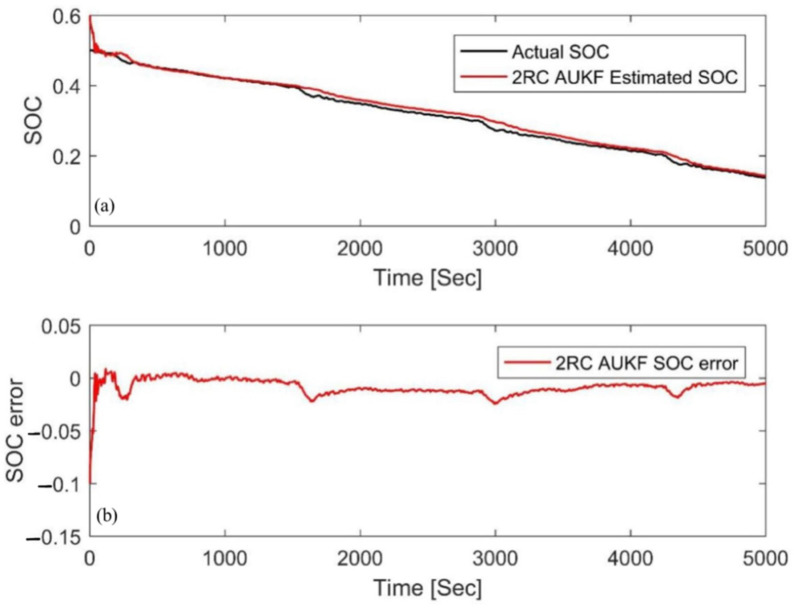
Estimation results at an initial SOC of 60% via the FUDS at 45 °C. (**a**) SOC estimation comparison curve; (**b**) SOC estimation error.

Figure 12 and Figure 13 are the estimation results and errors when the temperature was 45 °C and the initial value error was also ±10%, respectively. We can see that the initial errors can be compensated for quickly, and both of the MAE were 0.0091, as shown in Table 5. This result further shows that the AUKF has good temperature characteristics.

### 4.3. Simulation Comparison of Different Algorithms

**Figure 14:**
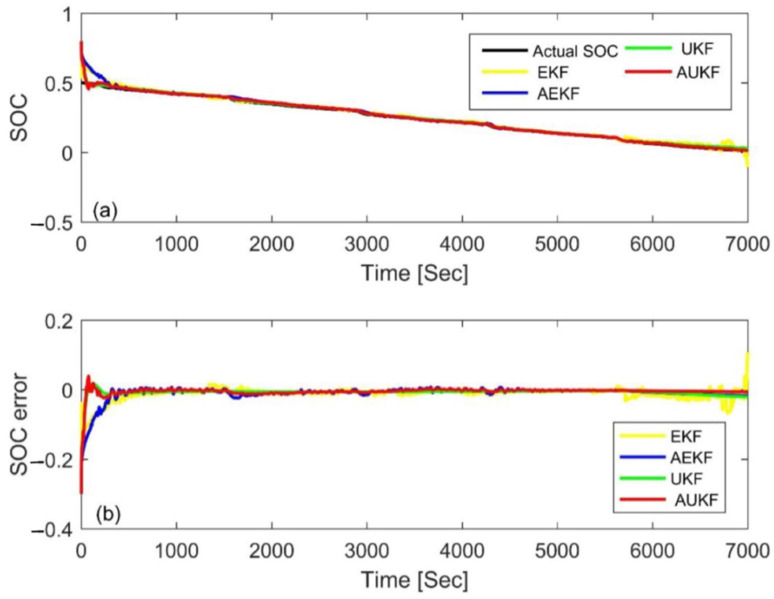
Estimation results at an initial SOC of 80% via the FUDS. (**a**) SOC estimation comparison curve; (**b**) SOC estimation error.

**Figure 15:**
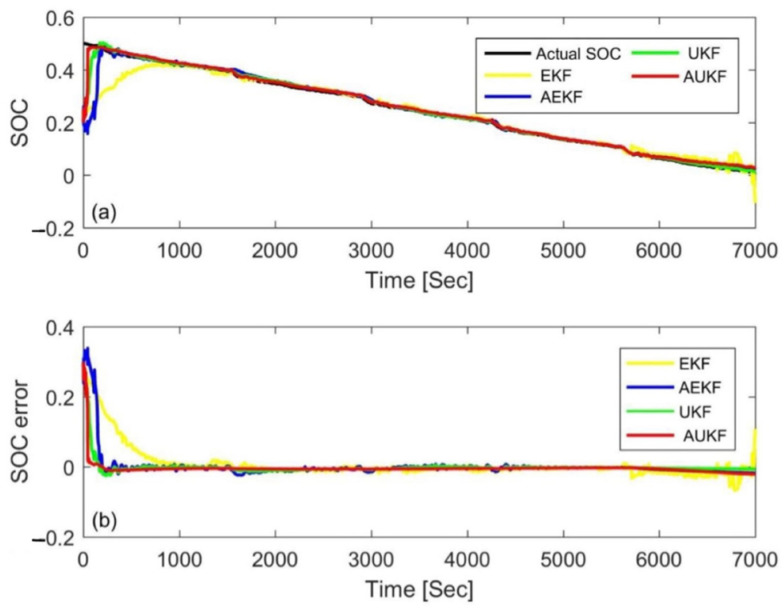
Estimation results at an initial SOC of 20% via the FUDS. (**a**) SOC estimation comparison curve; (**b**) SOC estimation error.

In order to further evaluate the performance of the AUKF, it was compared with the AEKF, EKF, and UKF algorithms in this paper. In the AEKF, the adaptive adjustment was realized via the covariance matching of residual voltage. The EKF and UKF use constant value of *Q_k_* and *V_k_* to estimate the SOC, and also use the trial and error method to determine the parameters. Figure 14 and Figure 15 show the estimation results and errors based on the four different Kalman filtering algorithms at an initial SOC of 80% and 20% under the FUDS cycle, respectively. In order to make the results more readable, the statistical table of the MAE and convergence time is shown in Table 6. Compared with the three other algorithms, the MAE value of the AUKF algorithm could be reduced by about one order of magnitude, and the convergence speed could be increased by an order of magnitude; therefore, it could estimate the SOC more quickly and accurately. Meanwhile, it could also be seen that the accuracy of the AUKF and AEKF was higher than that of the EKF and UKF. The results show that adaptive adjustment of the covariance difference between the process noise and the measurement noise can improve the estimation accuracy of the SOC. In addition, the UKF had better performance compared with the EKF. The comprehensive performance ranking of the four calculation methods was AUKF > AEKF > UKF > EKF.

**Figure 16:**
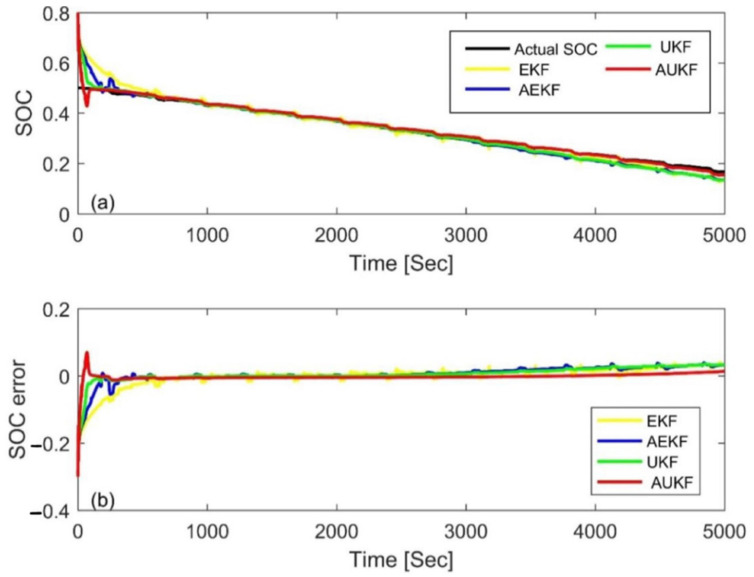
Estimation results at an initial SOC of 80% via the DST. (**a**) SOC estimation comparison curve; (**b**) SOC estimation error.

**Figure 17:**
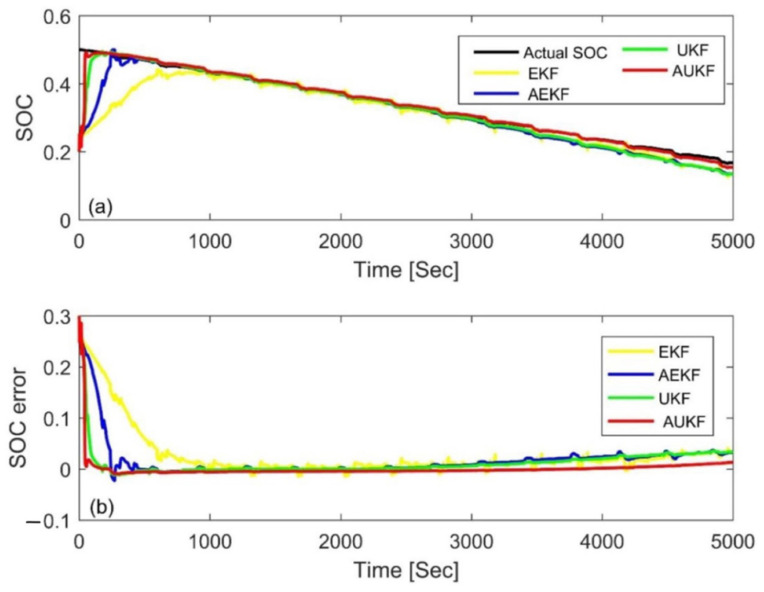
Estimation results at an initial SOC of 20% via the DST. (**a**) SOC estimation comparison curve; (**b**) SOC estimation error.

To further compare the performance of the four Kalman filter algorithms, the above four algorithms were applied to a DST cycle. Figure 16 and Figure 17 show the estimation results and errors based on different Kalman filtering algorithms with an initial SOC of 80% and 20%, respectively. Table 7 is the statistical table of the MAE and convergence time. From the results, one can see that the AUKF had the fastest convergence speed and the smallest MAE, which further confirms the superiority of AUKF. In addition, we also found that the EKF had the worst performance, while the UKF and AEKF had their own advantages. The UKF needed less calculation time but a larger mean absolute error, while the AEKF showed the opposite. This is different from the above result of the FUDS cycle. Nevertheless, the results further show that the idea of adaptive adjustment and nonlinear functions can improve computational accuracy.

## 5. Conclusions

In this paper, the AUKF was used to estimate the SOC of lithium-ion cells online, and its performance was evaluated systematically under large initial errors, wide temperature ranges, and different drive cycles. The results show that the estimation error was stable within 2%, and that the convergence speed was less than 50 s, which illustrates the excellent performance of the AUKF. Moreover, compared with the AEKF, EKF, and UKF, the AUKF was one order of magnitude smaller than that of the other three methods under the same initial value, and its mean absolute error was only 50% of that of the other methods. This is mainly because the AUKF not only has the advantage that UKF is better than EKF, but also extends the idea of covariance matching based on an output voltage residual sequence model to the UKF to realize adaptive regulation. In the process of the SOC estimation, the current SOC was continuously modified according to the mean and variance estimation results of each step of noise, which can correct the initial value error. Therefore, the AUKF had a better accuracy and a faster convergence speed than the other three algorithms, and can estimate the battery SOC more accurately and quickly.

Considering the identification accuracy and calculation time, this paper adopted the off-line exponential fitting method to identify the model parameters. If the identification accuracy needs to be further improved, the online identification method can be used to identify the battery model parameters in future work.

## Figures and Tables

**Table 1:** Basic specifications of the test samples.

Types	Positive and Negative	Rated Voltage	Rated Capacity	Up/Down Cutoff Voltage	Maximum Current
18,650	LiNiMnCoO_2_/ Graphite	3.6 V	2.0 Ah	2.5 V/4.2 V	22 A (25 Celsius)

**Table 2:** The battery model parameters.

*R*_0_ (Ω)	*R*_p1_ (Ω)	*C*_p1_ (F)	*R*_p2_ (Ω)	*C*_p2_ (F)
0.07898	0.009617	455.2766	0.012407	5573.927

**Table 3:** MAE and convergence rate at 25 °C.

Initial Value	MAE	Convergence Rate (s)
80%	0.0054	49
20%	0.0071	48

**Table 4:** MAE and convergence rate at 0 °C.

Initial Value	MAE
40%	0.0132
60%	0.0136

**Table 5:** MAE and convergence rate at 45 °C.

Initial Value	MAE
40%	0.0091
60%	0.0091

**Table 6:** Comparison results of the four calculation methods via the FUDS.

Algorithm	Initial Value	MAE	Convergence Time
EKF	80%	0.0134	489
EKF	20%	0.0212	725
UKF	80%	0.0075	55
UKF	20%	0.0081	140
AEKF	80%	0.0101	303
AEKF	20%	0.0123	169
AUKF	80%	0.0054	49
AUKF	20%	0.0071	48

**Table 7:** Comparison results of the four calculation methods via the DST.

Algorithm	Initial Value	MAE	Convergence Time
EKF	80%	0.0183	519
EKF	20%	0.0282	603
UKF	80%	0.0124	103
UKF	20%	0.0130	110
AEKF	80%	0.0151	167
AEKF	20%	0.0186	240
AUKF	80%	0.0056	36
AUKF	20%	0.0065	45

## Data Availability

The data presented in this study are available on request from the corresponding authors.
